# Linkage analyses identifiy novel loci for Alzheimer’s disease in non‐Hispanic white families

**DOI:** 10.1002/alz.092913

**Published:** 2025-01-03

**Authors:** Nicholas Gomez, Melissa Jean‐Francois, Kara L. Hamilton‐Nelson, Susan H. Slifer, Brian W. Kunkle, Michael L. Cuccaro, Eden R. Martin, Jonathan L. Haines, Christiane Reitz, Margaret A Pericak‐Vance, Badri N. Vardarajan, Gary Beecham

**Affiliations:** ^1^ John P. Hussman Institute for Human Genomics, University of Miami Miller School of Medicine, Miami, FL USA; ^2^ Department of Population and Quantitative Health Sciences, Institute for Computational Biology, Case Western Reserve University, Cleveland, OH USA; ^3^ G. H. Sergievsky Center, Vagelos College of Physicians and Surgeons, Columbia University, New York, NY USA; ^4^ 1501 NW 10th Avenue, Miami, FL USA; ^5^ Columbia University Irving Medical Center, New York, NY USA

## Abstract

**Background:**

Alzheimer disease (AD) is a progressive dementia with high heritability. While genome‐wide association studies have identified common variation associated with AD, most of these loci have effects too small to explain the segregation of disease within multiplex families. As such, these multiplex families likely harbor novel genetic variants with strong effects, and thus still play an important role in assessing the genetic etiology of AD.

**Method:**

Linkage analyses were conducted on 670 individuals across 70 non‐Hispanic white (NHW) multiplex families for AD or mild cognitive impairment (MCI). A combination of genome wide array data and imputed data was used for the analyses. Rare variants, and low‐quality variants were excluded; remaining variants were LD pruned. Linkage analyses included both multipoint (MPT) and two‐point (2PT) approaches, with non‐parametric, parametric (affected only; AO), and parametric (age‐penetrance; AP) models. Primary analyses included (1) all AD+MCI individuals and (2) all AD+MCI individuals with age at onset 65 or lower (early‐onset AD). Sensitivity analyses included the same models but excluding MCI.

**Result:**

We identified three primary linkage regions (HLOD > 3). For the MPT‐parametric‐AO model (AD+MCI) we identified chromosome 5q33 (HLOD = 3.017; 149MB‐156MB). A region on 8p11‐p21 (20MB‐37MB) was identified using the MPT‐parametric‐AO model (AD and early‐onset models; HLOD = 3.09, 3.27). Finally, chromosome 14q22‐24 (57MB‐70MB) was identified using the MPT‐parametric‐AP model (AD+MCI; HLOD = 3.56). The 5q33 region contains IL12B and ADAM19 genes. IL12B is an inflammatory cytokine that has been associated with amyloid burden and AD affection status. ADAM19 has been associated with AD progression and has functional links to APP. The 8p locus houses the CLU locus, first identified in large AD GWAS, while the 14q locus is near the PSEN1 gene. Further analyses of whole genome sequence data in these regions are ongoing.

**Conclusion:**

These analyses highlighted three regions and several genes connected with Alzheimer’s disease. These new loci, along with the established loci identified, may give more insight into the causes of AD for multiplex families.

